# Tracking Knowledge Evolution in Cloud Health Care Research: Knowledge Map and Common Word Analysis

**DOI:** 10.2196/15142

**Published:** 2020-02-25

**Authors:** Dongxiao Gu, Xuejie Yang, Shuyuan Deng, Changyong Liang, Xiaoyu Wang, Jiao Wu, Jingjing Guo

**Affiliations:** 1 The School of Management Hefei University of Technology Hefei China; 2 The Seidman College of Business Grand Valley State University Grand Rapids, MI United States; 3 The 1st Affiliated Hospital Anhui University of Traditional Chinese Medicine Hefei China; 4 College of Business Administration Central Michigan University Mount Pleasant, MI United States

**Keywords:** cloud health care, cloud computing, health care informatics, cybermetrics, co-word analysis

## Abstract

**Background:**

With the continuous development of the internet and the explosive growth in data, big data technology has emerged. With its ongoing development and application, cloud computing technology provides better data storage and analysis. The development of cloud health care provides a more convenient and effective solution for health. Studying the evolution of knowledge and research hotspots in the field of cloud health care is increasingly important for medical informatics. Scholars in the medical informatics community need to understand the extent of the evolution of and possible trends in cloud health care research to inform their future research.

**Objective:**

Drawing on the cloud health care literature, this study aimed to describe the development and evolution of research themes in cloud health care through a knowledge map and common word analysis.

**Methods:**

A total of 2878 articles about cloud health care was retrieved from the Web of Science database. We used cybermetrics to analyze and visualize the keywords in these articles. We created a knowledge map to show the evolution of cloud health care research. We used co-word analysis to identify the hotspots and their evolution in cloud health care research.

**Results:**

The evolution and development of cloud health care services are described. In 2007-2009 (Phase I), most scholars used cloud computing in the medical field mainly to reduce costs, and grid computing and cloud computing were the primary technologies. In 2010-2012 (Phase II), the security of cloud systems became of interest to scholars. In 2013-2015 (Phase III), medical informatization enabled big data for health services. In 2016-2017 (Phase IV), machine learning and mobile technologies were introduced to the medical field.

**Conclusions:**

Cloud health care research has been rapidly developing worldwide, and technologies used in cloud health research are simultaneously diverging and becoming smarter. Cloud–based mobile health, cloud–based smart health, and the security of cloud health data and systems are three possible trends in the future development of the cloud health care field.

## Introduction

“Cloud” is a metaphor for the internet in the information age. Through a computer network, cloud computing provides scalable, distributed computing solutions that greatly alleviate problems related with a lack of computing power in various fields [1]. Largely considered as the next revolution in information technology (IT), cloud computing has become one of the most researched topics among IT scholars since 2007. The concept of cloud computing was introduced to the IT research community in China in 2008, after which it quickly became popular. The nation’s Twelfth Five-Year Plan recognized cloud computing’s strategic position in the IT industry [2]. In the meantime, major internet companies such as Google, IBM, Amazon, and Microsoft have invested considerable resources to develop and promote their cloud computing services. Driven by both national policies and industry advances, scientific research on cloud computing has experienced tremendous growth. The number of scientific articles related to cloud computing has increased from 982 in 2007 to more than 20,000 as of December 2017 (based on the Web of Science database). In addition to the development of technology, the idea of “cloud” has been applied to a variety of applications such as cloud health care, cloud education, and cloud life.

In the area of health care, cloud computing has played an increasingly important role in providing storage and computing power for the massive volumes of data [3]. In the age of big data, the development of the Internet of Things (IoT) and sensor networks has spurred the growth of medical and health data. At the same time, IT has been universally used to support diagnosis as well as medical and health information management thanks to its potential strategic and financial impacts. In this trend, a new application of cloud computing in health care has been created and given the name “cloud health care.” Cloud health care refers to health services that improve the delivery of diagnosis and treatment by more efficiently utilizing medical resources through technologies such as cloud computing and IoT [4].

Researchers and practitioners worldwide have extensively explored cloud health care. There are many studies in health care that are based on cloud computing. For example, He et al [5] developed a robust, reliable, and efficient cloud platform architecture. It can meet a high number of concurrent requests from ubiquitous health care services because of the pervasive and on-demand, service-oriented nature of cloud computing. Wang et al [6] designed and evaluated a mobile health information system based on cloud computing and wireless sensor networks; they adopted the gray theory and Markov model to predict the moving path of objects jointly. Fong and Chung [7] suggested the use of mobile cloud computing for a health care system. Mobile devices were used as terminals that allowed medical professionals and family members to easily access medical data. Antypas et al [8] showed a positive effect of an internet- and mobile phone–targeted intervention on physical activity for patients with cardiovascular disease. Cheung [9] analyzed data from patients who participated in the National Health and Nutrition Survey via mobile medical testing centers. In July 2011, Zhongxing Telecommunication Equipment Corporation, a leading Chinese telecommunication company, launched the Healthy Cloud Healthcare program. It uses wireless medical equipment to collect human health data in real time and uploads the data to the cloud service system. The system can maintain electronic medical records for individuals. Users can access data through mobile wireless devices such as mobile phones. In this way, they can not only understand their health conditions at any time and from any location but also maintain timely interactions with their physicians through the platform.

Despite the abundant body of literature on cloud health care, little effort has been made to curate and refine the knowledge from the literature. First, there is a lack of understanding of the evolution of global knowledge on cloud health care. Second, research hotspots in cloud health care have not been identified. Third, the future trends in cloud health care are not understood. After a decade of exploration, it is important to understand the current status of cloud health care research and how it will develop in the future. Therefore, drawing on the cloud health care literature, this study aimed to identify the development and evolution of research themes in cloud health care through a knowledge map and common word analysis.

This article contributes to the cloud computing medical research community by summarizing research achievements and identifying new frontiers of research. Literature about science and technology represents one of the most comprehensive and concrete manifestations of scientific research results. It is an important tool for researchers to exchange ideas and communicate research findings. Electronic journal databases have made it possible and convenient to find and collect a large amount of literature [10]. In-depth analysis of science and technology literature is a common approach to understanding the state of research in a field. Both quantitative and qualitative analyses of the literature in the field of cloud health care can help describe the discipline’s research status and accomplishments, help researchers understand the extent of research development in cloud health care to inform future studies, and provide an important reference for further contributions to the field. From a practical point of view, these analyses also support health care practitioners in translating research findings into industry solutions, potentially reducing medical costs and increasing the utilization of medical resources.

The rest of the paper is organized as follows: presentation of data and research methods and tools; reporting of the findings from the knowledge evolutionary analysis, co-word analysis, and sudden word analysis of the cloud health care literature; and a discussion of the implications to cloud health care research.

## Methods

### Data Collection and Preprocessing

The Web of Science, developed by US Thomson Scientific, includes three cited libraries: Science Citation Index, Social Sciences Citation Index, and Arts & Humanities Citation Index. It also includes two chemical databases: Current Chemical Reactions and Index Chemicus [11]. It contains not only articles published in academic journals but also other types of publications such as conference proceedings and patents. Using the database, one can retrieve relevant literature titles and summaries, the references used in each paper, and the context in which each paper was cited [12].

**Figure 1:**
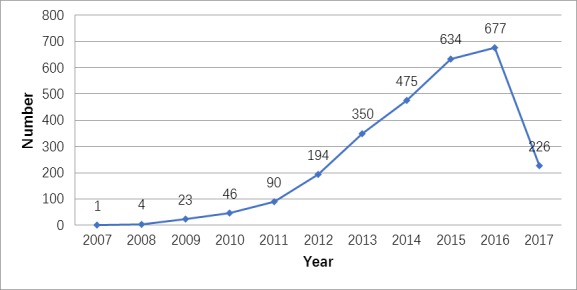
The number of articles related to cloud health care, by year.

This study used the following as data sources: Science Citation Index Expanded, Conference Proceedings Citation Index-Science, Current Chemical Reactions Expanded, and Index Chemicus. On August 14, 2017, we retrieved relevant articles published between 2007 and 2017. In this paper, the following two search types were used with the “AND” relationship in the advanced search method: TS = (hospital * OR heart * OR blood * OR disease * OR medical * OR #) (“#” represents the 20 keywords related to the health sector) and TS = (“cloud comput *” OR “SaaS” OR “PaaS” OR “IaaS” OR ##), where “##” represents more than 11 keywords related to cloud computing. The search returned 2878 articles; the types included “proceedings paper,” “article,” and nine other types. According to the search results, articles related to cloud health care were scarce before 2010. At the beginning of 2010, the number of related articles began to rapidly increase. By 2016, the number of articles had increased by nearly 10 times that in 2010 (Figure 1). Therefore, cloud health care has become a hot topic in recent years.

To establish a sequential word network, the bibliographic information was divided into four periods: 2007-2009 (Phase I), 2010-2012 (Phase II), 2013-2015 (Phase III), and 2016-2017 (Phase IV). Terms synonymous with different forms of the term were replaced. For example, “Internet of Things” uniformly replaced “IoT” and “internet of things.” Subsequently, a symmetric co-occurrence matrix was generated by counting the co-occurrences of the two keywords. The data in the diagonal cell was treated as the key word frequency, and the value of the non-diagonal cell was the common word frequency [13].

### Data Analysis Methods and Tools

This study used keywords to analyze the relevant research articles about cloud health care. Keywords are the core concepts in the study of natural language forms. Keywords can be used to distinguish the contexts and methods of research articles. Therefore, multiple time series of keywords extracted from research papers in a certain field can reveal the development and trend of research in that field [14]. For statistical modeling and data visualization purposes, we used the Statistical Analysis Toolkit 3.2 (SATI; Liu Qiyuan and Ye Ying, China), Network Evolvement and Trend Detection System 1.5 (NEViewer; Wuhan University, Wuhan, China), UCINET 6.186 [15], Python 2.7 (Amsterdam, Netherlands), and NetDraw 2.084 [16].

To visualize the evolution of keywords in cloud health care, we used NEViewer [17]. The data structure used in NEViewer is a sequential word network. The Python program was used to store the co-word network of each period as an nwb format file, and thematic evolution was probed after loading the file in NEViewer. NEViewer shows the macroscopic evolutionary process and microscopic evolution details of the online community in a novel way using an alluvial graph and a color rendering network diagram, which is used by many scholars.

In this study, we used SATI to extract keywords and get a 100-by-100 co-occurrence matrix of high-frequency words in each phase. The co-occurrence matrix was ​​the basis for further analysis. SATI is an article title statistical analysis tool with four functions: title format conversion, field information extraction, entry frequency statistics, and knowledge matrix construction [18]. Subsequently, we used UCINET and NetDraw to draw and analyze keyword co-occurrences. UCINET is a powerful social networking analytics software that does not include graphical visualization but allows data to be exported and processed to software such as NetDraw, Pajek, Mage, and KrackPlot [19]. UCINET can handle the raw data matrix format. Therefore, the co-occurrence matrix was ​​generated based on SATI 3.2. Then, the co-occurrence matrix was ​​introduced into UCINET for analysis. Finally, we used NetDraw to produce a knowledge map to visualize the interrelationship of the keywords, making it easy to learn and analyze.

## Results

### Knowledge Evolution Analysis

**Figure 2:**
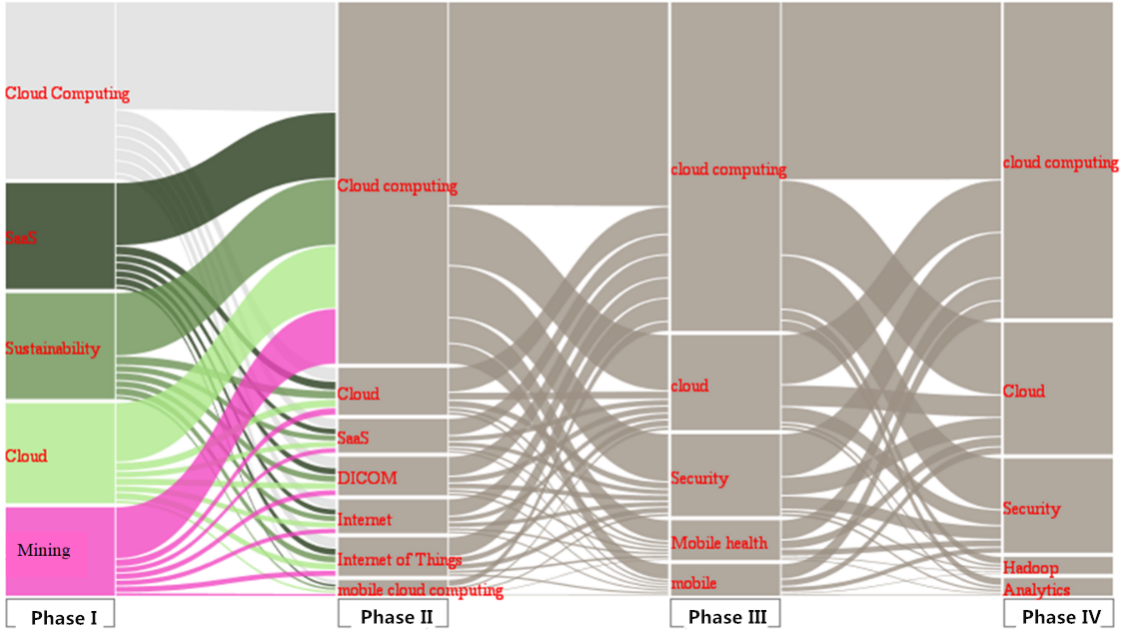
Cloud health care knowledge evolution map.

NEViewer functions include topic clustering and community division. It can be applied to the analysis of evolutionary processes of many complex networks such as social network evolution, knowledge network evolution, enterprise network evolution, and human network evolution. The processed file was imported into NEViewer for Blondel community detection processing, and the evolving situation of foreign hotspot communities in various periods is shown in Figure 2. The rectangular color block represents the community, the curved color patches between the 2 time segments represent the evolution process, and the height of the color patches represents the community node size [20]. Using these theme communities, we can visualize the evolution of hotspots, show the hotspots, and show the changing themes in cloud health care research.

Figure 2 shows the 4 major hotspot communities categorized by the 4 phases. It also shows a map of changes and the evolution of the hotspot communities. The rectangular color block area indicates the criticality of the elements it contains and the amount of research performed about the subjects in the community; a larger area signifies greater criticality and more research. In Phase I, the top 5 keywords ranked by community size were “Cloud Computing,” “software as a service (SaaS),” “Sustainability,” “Cloud,” and “mining.” In Phase II, the top 5 keywords were “Cloud Computing,” “Cloud,” “SaaS,” “Digital Imaging and Communications in Medicine (DICOM),” and “Internet.” In Phase III, the top 5 keywords, from highest to lowest in terms of community size, were “Cloud Computing,” “Cloud,” “Security,” “Mobile health,” and “mobile.” In Phase IV, the top 5 keywords ranked by community size were “Cloud Computing,” “Cloud,” “Security,” “Hadoop,” and “Analytics.”

The evolution of the knowledge network includes the generation, disappearance, division, and merger of knowledge. The evolutionary manifold of NEViewer can vividly show the process of extinction, differentiation, and integration of hotspot community evolution. Using “Cloud Computing” as an example, from Phase I to Phase II, “mining” differentiated into “Cloud Computing” and “Internet.” From Phase II to Phase III, “Cloud Computing” differentiated into “Security” and “Mobile health.” Mobile health became a new research hotspot in Phase III. From Phase III to Phase IV, “Cloud Computing” differentiated into “Cloud” and “Security” as well as “Hadoop” and “Analytics,” among others. This shows that the research scope of cloud computing in the medical field is constantly expanding and deepening. Meanwhile, “Mobile health” merged with “Cloud Computing” and “Cloud.” In addition, from Phase I to Phase II, “Cloud Computing” differentiated into “Internet of Things” and “mobile cloud computing.” From Phase II to Phase III, “Internet of Things” merged with “mobile.” From Phase III to Phase IV, “mobile” merged again with “Cloud Computing.” Therefore, the evolution and development of the cloud health care field was firmly centered on cloud computing.

### Co-Word Analysis

As a refined way to express a subject in academic publications, the correlation between keywords can reveal the inherent relationship of knowledge in the academic field, to a certain extent. Co-word analysis is similar to co-citation or co-occurrence analysis [21] and is considered a reasonable method to describe the relationship between concepts, perspectives, and problems [22]. In co-word analysis, it is assumed that the keywords extracted from an article can represent a specific research direction, research topic, or topic of a field. If two keywords appear together in one article, then the two topics they represent are related. The higher the co-word frequency, the stronger the relationship between keywords, which further indicates that the two keywords are related to a specific research topic [23].

We constructed co-word matrices by keywords and their co-occurrence relationships and then mapped them to a co-word network. The distance between the nodes in the network reflects the relationship between the topic content. The co-word network, when combined with time, can reflect the evolutionary trend of the scientific literature and the development process of the entire discipline. The co-occurrence and evolution analyses of the keywords can highlight updates in scientific research topics and capture the patterns of knowledge production.

Co-word analysis has been applied in various fields of research. This paper explores the main research directions and hotspots in the cloud health care field in the four phases from 2007 to 2017 through common word analysis and discusses the trends for future development.

### Co-Word Analysis for Phase I (2007-2009)

**Figure 3:**
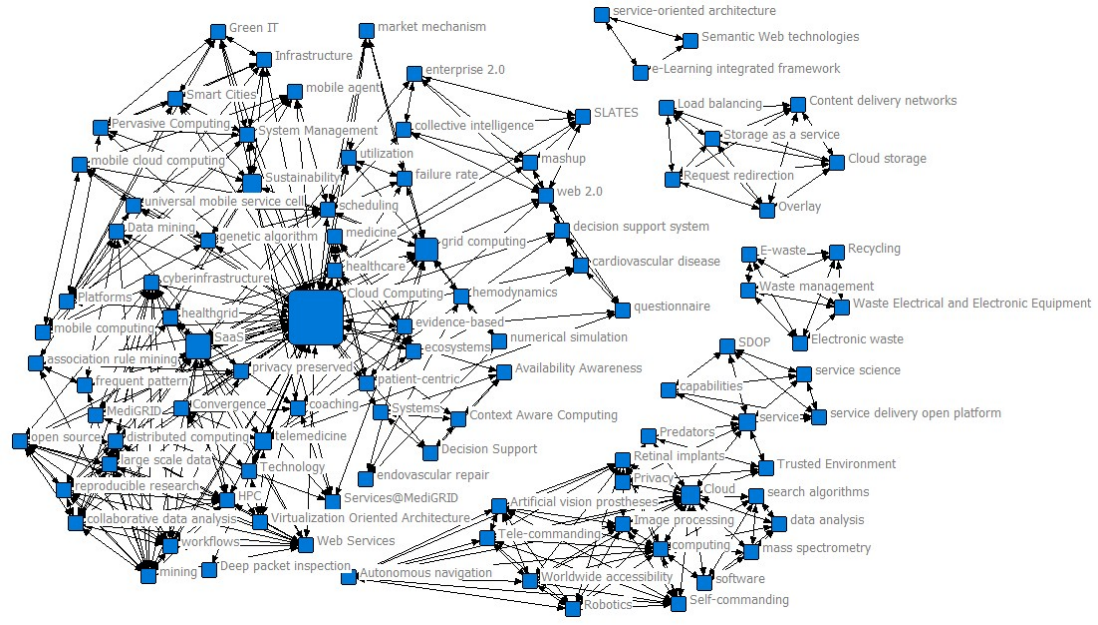
Keyword co-occurrence map for 2007-2009.

The size of the node characterizes betweenness of the keywords. The larger the nodes, the more the keywords are related to each other and the more likely it is to expand to the keywords of other research topics [24]. As shown in Table 1 and Figure 3, “Cloud Computing,” “SaaS,” and “grid computing” had significantly strong betweenness in Phase I. In addition to “Cloud Computing,” “grid computing” primarily linked the cloud and medical research community. Currently, grid computing technology provides a just-in-time service for users by sharing and collaborating on all the resources (ie, computing, storage, communications, information resources, and knowledge resources) on the internet [25]. In addition, “telemedicine” had a strong central agency. It shows that telemedicine first appeared in the field of cloud health care treatment as a type of medical technology. Telemedicine is a medical technology that integrates medicine, computer technology, and communication technology. In 2006, the United Kingdom invested more than 170 million British pounds in telemedicine research [26]. The development of telemedicine marks the convergence of the health care industry and cloud computing. In this phase, the frequencies of co-occurring words are relatively low, which shows that cloud health care treatment was in its infancy.

Watson et al [27] developed a common electronic science platform in the cloud that supports metadata sharing, integration, and analysis. Scientists built on this systematic study to understand how the brain works, an area representing both opportunities and challenges in biology, medicine, and computer science. Sundararaman et al [28] presented a SaaS-based application, Hridaya, that, in conjunction with devices such as personal digital assistants and mobile phones, allows patients to report their health to a doctor on a schedule. Subsequently, doctors can monitor patients to improve recovery and survival. Bauer and Mohtashemi [29] proposed a parallel Monte Carlo model using cloud computing as a method to not only meet real-time monitoring constraints in the medical field but also reduce or eliminate the costs associated with real-time disease monitoring systems. Meir and Rubinsky [30] introduced a new paradigm of medical technologies focusing on wireless technologies and cloud computing that were designed to overcome the growing cost of medical technology. This new paradigm also allows untrained medical staff to perform imaging and generate more accurate diagnoses to more effectively save patients’ lives.

Medical technology is indispensable to modern medicine. However, in the case of an influx of data, it becomes very expensive and complex and therefore inaccessible. Therefore, in Phase I, most scholars applied cloud computing to the medical field mainly to solve high-cost problems. In recent years, cloud computing has accelerated the construction of medical information resources, realized the sharing of information resources, improved the service level of medical institutions, and reduced the cost of building medical information systems.

**Table 1:** Betweenness of the top 10 keywords in Phase I.

Keywords	Betweenness	Frequency
Cloud computing	1446	9
SaaS	344	3
Grid computing	286	3
Sustainability	121	2
Cloud	120	3
Telemedicine	99	2
Service	68	2
Mining	33	2
Scheduling	24	2
Computing	16	2

### Co-Word Analysis for Phase II (2010-2012)

**Figure 4:**
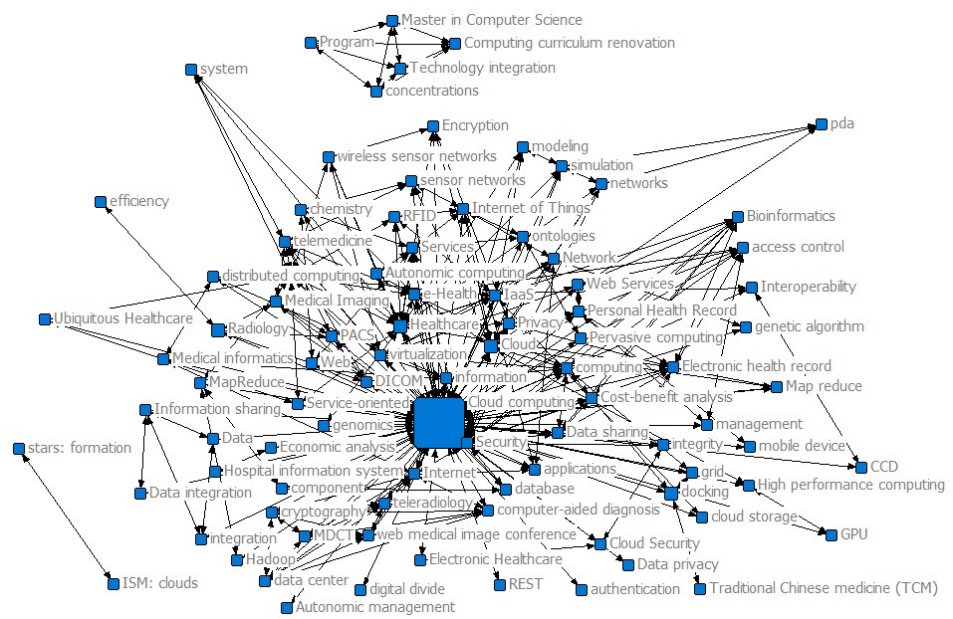
Keyword co-occurrence map for 2010-2012.

If a node in a network has strong relationships with other nodes, it has high centrality and is very important in the network. In Phase II, “Cloud Computing” had the highest level of betweenness and occupied an important position, becoming the largest bridge among the other research hotspots. In addition, the levels of betweenness of “Cloud” and “Healthcare” were also strong. The emergence of “Healthcare” shows that the research topics in the field of medicine were extensively explored and that other emerging research points are likely to be expanded through those topics. Studying the high-frequency keywords in a certain field helps to determine the context development, hotspots, and trends. As shown in Table 2 and Figure 4, “Cloud Computing” had a high frequency, one that was far greater than that of the other popular keywords “Healthcare,” “Security,” “Cloud,” “SaaS,” and “Personal Health Record.”

**Table 2:** Betweenness of the top 15 keywords in Phase II.

Keywords	Betweenness	Frequency
Cloud computing	3462	144
Healthcare	134	19
Cloud	100	14
Docking	91	3
Radiology	91	2
Security	72	19
SaaS	65	10
Personal health record	41	9
Networks	38	3
eHealth	36	6
Simulation	36	3
Telemedicine	29	8
Bioinformatics	28	4
Computing	23	5
High-performance computing	23	3

During this period, radiology led the promotion of technology development, such as cloud computing, in the medical field, which might be attributed to the relatively rich IT-related knowledge of radiologists. Krestin et al [31] reported that radiologists were the first to promote integrated diagnostics through IT solutions and cloud computing. Satoh et al [32] developed a new remote radiology network system based on information security solutions to improve the speed and accuracy of diagnoses and to enhance the protection of personal information. Koufi et al [33] embedded context-aware access control into eRadiology workflows and worked with personal health record systems that were already implemented in cloud computing infrastructures. The resulting security system ensured precise and tight access control.

In view of this, compared with Phase I, in which scholars focused on expanding data volumes and high costs using grid computing, cloud computing, and other technologies, Phase II saw scholars paying attention to system security issues. Implementing cloud computing in the day-to-day medical operations has many benefits. However, health care organizations and employees have encountered resistance to this modern technology due to patient data privacy and security issues. To take full advantage of the power of cloud computing, comprehensive and secure solutions for cloud processing are needed. Nguyen et al [34] introduced a cloud service system model and a security mechanism based on key management with secure multicast. Feldman et al [35] designed and developed a novel method to protect personal medical information in a remote and physically unsustainable environment.

### Co-Word Analysis for Phase III (2013-2015)

**Figure 5:**
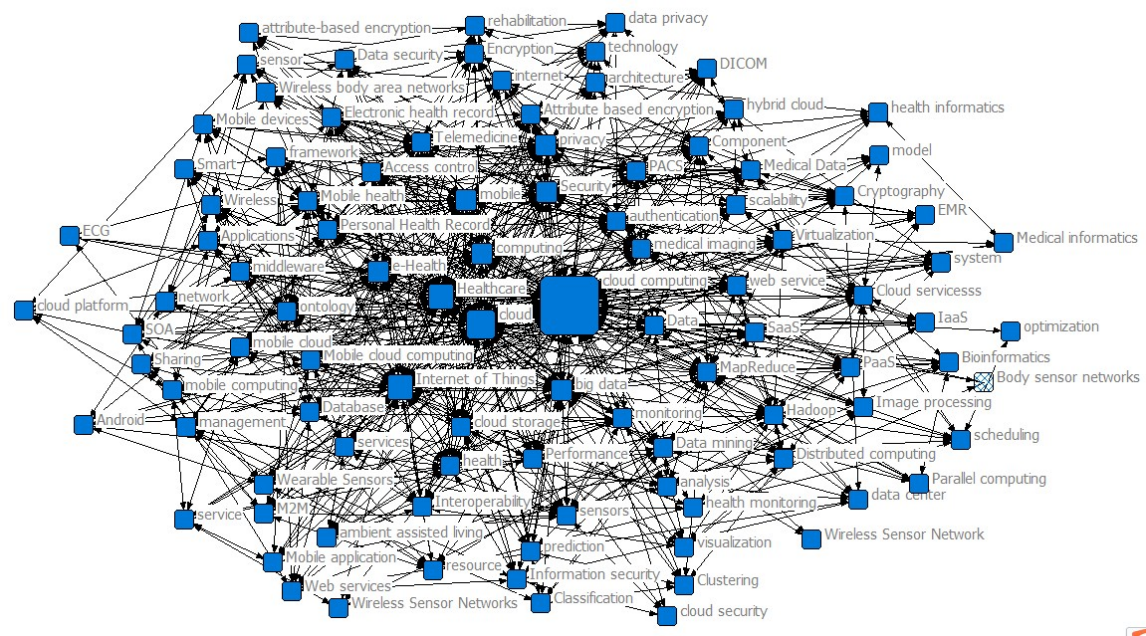
Keyword co-occurrence map for 2013-2015.

In Phase III, “Cloud Computing” had the strongest betweenness, followed by “Cloud,” “Healthcare,” “Internet of Things,” “big data,” “Security,” and “eHealth.” As shown in Figure 5*,* the betweenness of “Cloud Computing” remained at the top of the list. However, its drop from the previous higher ranking might be related to the emergence of bridges such as “Internet of Things” and “big data” in this phase, which weakened the role of “Cloud computing” as a liaison.

With the rapid development of computer science and IT, health care informatization has been widely implemented, generating massive volumes of data in health care and health management. The development and application of big data in health care play an important role in promoting medical and health services and the development of healthy industries [36]. In the era of big data, cloud computing is the core of IoT. At the same time, it can promote the intelligent convergence of the IoT and the internet. IoT refers to an open and comprehensive network of intelligent objects that have the capacity to auto-organize; share information, data, and resources; and react and act to situations and changes in the environment [37]. It was estimated that 1 billion devices would be connected to the internet by 2020 [38]. In the field of health care, IoT has played an increasingly important role and is widely used [39]. A wide range of applications have been developed based on the IoT. Cubo et al [40] designed and implemented a cloud–based IoT platform to help health care professionals seamlessly monitor the health of many patients and data in real-time. From the perspective of health care systems engineering, Fernandez and Pallis [41] analyzed the opportunities for and challenges of one health IoT platform to strengthen its medical services, clinical care, and remote monitoring. Skraba et al [42] developed a prototype speech-controlled, cloud-based wheelchair platform for disabled persons.

More specifically, scholars have proposed algorithms and architectures to address efficiency and security issues. Nakagawa et al [43] proposed m-cloud, a distributed computing mechanism with multiple cloud resources. It not only increases resiliency, scalability, and efficiency but also reduces the risk of revealing private information. Kim [44] proposed a polynomial-time algorithm to download energy-saving dynamic packets from medical cloud storage to medical IoT equipment. Suciu et al [45] proposed an architecture for eHealth applications that integrate big data, IoT, and the cloud. Mital et al [46] developed a framework of cloud computing–based smart community services and the emerging cloud computing ecosystems. This research has wide-ranging implications on the future of the IoT and can be extended to elderly health and support, energy-efficient systems, and smart cities. In the future, smart cities, smart homes, wearables, and mobile computing will become frontiers in the development of the IoT [47].

eHealth is an emerging area at the intersection of medical informatics, public health, and commerce. It involves health services and information provided or enhanced through the internet and related technologies. With the rapid development in such fields as cloud computing and big data, more extensive research attention will occur for eHealth. At present, the widespread application of electronic health records has led to the rapid accumulation of medical data. Big data analytics can extract this knowledge to improve the quality of health care, which is also an important reason for the rise of “big data” as a keyword in the co-word network [48]. In addition, since the emergence of “Security” in Phase II, it has received sustained attention from scholars. With the continuing popularization of cloud computing, the importance of security is attracting more attention. Especially in the medical field, security has also become the most important concern for patients.

Table 3 lists the top 15 keywords and their characteristics in Phase III. The high-frequency keywords included “Cloud Computing,” “Cloud,” “Healthcare,” “Internet of Things,” “Security,” “big data,” and “eHealth”. These keywords represent hotspots in the cloud computing and medical areas. Compared with previous periods, the frequency of keywords greatly increased, demonstrating that scholars gradually increased and deepened their research on cloud health care services during this period.

**Table 3:** Betweenness of the top 15 keywords in Phase III.

Keywords	Betweenness	Frequency
Cloud computing	1921	447
Cloud	511	128
Healthcare	256	74
Internet of Things	247	67
Big data	140	58
Security	122	66
eHealth	89	57
Computing	73	41
Telemedicine	73	23
Privacy	68	42
MapReduce	40	23
Ontology	33	14
Health	25	13
Personal health record	24	28
Mobile cloud computing	24	23

### Co-Word Analysis for Phase IV (2016-2017)

**Figure 6:**
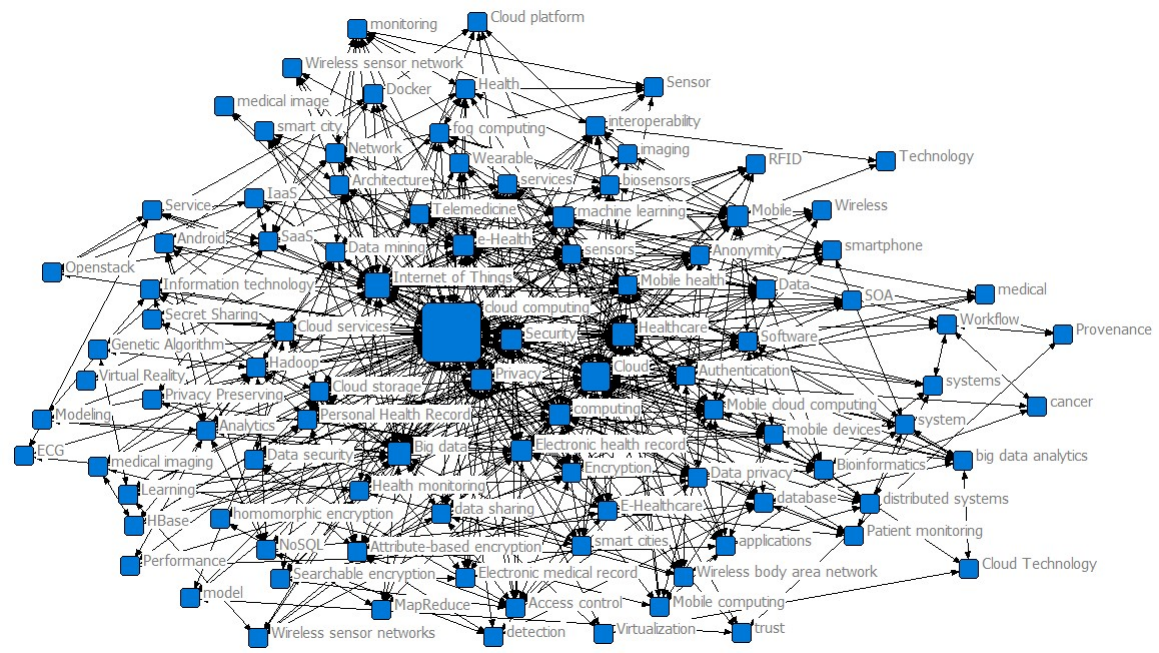
Keyword co-occurrence map for 2016-2017.

As shown in Table 4 and Figure 6, in Phase IV, the most betweenness centralities were “Cloud Computing,” “Cloud,” “Internet of Things,” “Healthcare,” “big data,” “eHealth,” “computing,” “Security,” and “machine learning.” Compared with Phase III, “Internet of Things,” “big data,” and “eHealth” increased as keywords, and “machine learning,” “mobile,” and “Mobile health” emerged as hotspots.

In this phase, machine learning gradually drew health care scholars’ attention. As an example, natural language processing has been used to structure electronic health records [49]. Classification and prediction methods have also been used for computer-aided diagnosis. Santillana et al [50] proposed a machine learning model for real-time influenza estimation. Gupta et al [51] assembled three models of Naive Bayes, AdaBoost, and boosted tree methods to improve the accuracy of heart disease prediction. Gu et al [52] presented a case-based reasoning system for breast cancer–related diagnoses. In addition, algorithms such as decision trees and neural networks are used for image classification and disease classification [53,54]. We summarized the relevant literature in Table 5. Machine learning algorithms have been widely used in the medical field during Phase IV. However, from the perspective of efficiency, the algorithm accuracy needs improvement. In the future, suitable machine learning methods will be used to increase efficiency.

**Table 4:** Betweenness of the top 15 keywords in Phase IV.

Keywords	Betweenness	Frequency
Cloud computing	2167	252
Cloud	532	66
Internet of Things	313	77
Healthcare	257	38
Big data	206	52
eHealth	150	30
Computing	128	28
Security	119	34
Machine learning	87	14
Privacy	71	29
Electronic health record	71	13
Mobile	63	11
Sensors	38	10
Authentication	28	13
Mobile health	26	15

**Table 5:** Summary of the relevant literature regarding machine learning in cloud health care.

Objective	Algorithm	Efficiency	Source
To introduce a double-reading entry system for extracting clinical data from unstructured medical records and creating a semistructured electronic health record database	Natural language processing	This reproducibility study with magnetic resonance images from 100 patients had an overall high reproducibility of 98%.	Luo et al [49]
To build a machine learning model named AutoRegressive Electronic Health Record Support Vector Machine (ARES) to provide real-time influenza estimates	Support vector machine	ARES can estimate national influenza-like illness activity with an almost tenfold reduction in the average error.	Santillana et al [50]
To provide a practical, comprehensive workflow for typical machine learning problems seen in medical image analysis	Binary classification, multi-class learning, regression	The benchmark was conducted on 14 public data sets and 4 local medical image data sets using a single common flow. It ensured better (similar to 8% improvement) or at least similar generalization capability with respect to existing methods.	Roychowdhury et al [52]
To find optimal image-based feature sets that reduce computational time complexity while maximizing overall classification accuracy	Decision tree	It significantly enhanced the borderline classification performances in automated screening systems.	Roychowdhury et al [53]
To distinguish between the gait features of a person with Friedreich's ataxia and a person with healthy gait characteristics	Neural network (multilayer perception)	A considerable degree of classification accuracy was achieved. The attributes of the gyroscope signals (roll and yaw) attained classification accuracies of 74% and 63%, respectively.	LeMoyne et al [54]
To predict heart disease	Naive Bayes, AdaBoost, boosted tree	The assembled model increased the overall accuracy to 87.91%.	Gupta et al [51]

In Europe, the United States, and other countries, the mobile health business has moved into the service phase. For example, portable sensing terminals that measure parameters such as electrocardiograms, blood glucose levels, and blood pressure have provided convenient methods for both doctors and patients. With the rise of the mobile internet, medical health applications such as Chunyu Doctor, Baidu, and Pomelo are increasingly used by the general public. As a result, mobile health and mobile medical services have become hotspots among scholars worldwide. Witbrodt and Sunderam [55] proposed a patient-centric mobile medical cloud platform for real-time data collection and monitoring. They also proposed a cloud storage solution for mobile data flows. The acquisition of modern medical information, especially in rural areas of developing countries, is crucial for effective health care. For instance, Miah et al [56] designed and evaluated an innovative mobile decision support system to address such issues as health decision support and information dissemination for farmers.

To further understand the research hotspots throughout all the studied years, we analyzed and summarized the keywords of 2007-2017. Table 6 provides the overall distribution of the co-occurrence of the keywords through the central table of intermediaries. The top 5 keywords in the intermediary were “Cloud Computing,” “Cloud,” “Healthcare,” “Internet of Things,” and “Security.” This is largely consistent with the popular words in recent years. Table 7 summarizes the keywords and main findings of each phase.

**Table 6:** Betweenness of the top 15 keywords in 2007-2017.

Keywords	Betweenness	Frequency
Cloud Computing	1127	852
Cloud	459	211
Healthcare	334	132
Internet of Things	236	148
Security	154	119
eHealth	146	93
Big data	145	111
Computing	134	76
Privacy	92	78
Mobile cloud computing	79	39
Mobile	74	35
Electronic health record	53	44
SaaS	52	41
Data	40	25
Health	40	23

**Table 7:** Keywords and main findings of each phase.

Phase	Keywords	Discovery
I (2007-2009)	Cloud Computing, SaaS, grid computing, Sustainability, Cloud, telemedicine, service, mining, scheduling, computing	Most scholars applied cloud computing to the medical field mainly to reduce cost, and most of the technologies were based on grid computing and cloud computing.
II (2010-2012)	Cloud Computing, Healthcare, Cloud, docking, radiology, Security, SaaS, Personal Health Record, Networks, eHealth, Simulation, telemedicine, Bioinformatics, computing, high-performance computing	Scholars began to focus on cloud-based security issues, including patient data privacy and security.
III (2013-2015)	Cloud Computing, Cloud, Healthcare, Internet of Things, big data, Security, eHealth, computing, telemedicine, privacy, MapReduce, ontology, health, Personal Health Record, mobile cloud computing	With the rapid development of computer science and information technology, health care informatization was widely used. Mass data sets formed big data in health care. The Internet of Things rapidly developed.
IV (2016-2017)	Cloud Computing, Cloud, Internet of Things, Healthcare, big data, eHealth, computing, Security, machine learning, Privacy, Electronic health record, mobile, Sensors, Authentication, Mobile health	Machine learning and mobile technology were introduced into the medical field.

### Sudden Word Analysis

The identification and tracking of research frontiers can inform researchers of the evolution of research topics, forecast the development of research, and identify problems that need to be further explored [57]. Chen believes that a research frontier is an emerging theoretical trend and research theme that can be expressed by the sudden increase in technical terminology (ie, sudden words) [58]. In this study, the first 100 keywords of each phase were compared. When certain keywords did not exist in the previous phase but appeared in the current phase, they were considered as the emergent words of the current phase. In this study, sudden words were identified using the Python program for Phases II, III, and IV (Table 8).

In Phase II, most of the sudden words were also high-frequency keywords in that period. This indicates rapid development of the cloud health care field during this phase. Emerging keywords such as “Electronic health record,” “Personal Health Record,” and “DICOM” suggest that storage technology was used to solve the problem of data expansion that came with the adoption of personal health records and digital medical images. For example, Fernández-Cardeñosa et al [59] proposed two solutions for electronic health record systems based on cloud computing. Radwan et al [60] also presented a cloud-based platform that provides developers, health care providers, and organizations with a framework for retrieving and managing medical records and personal health records.

**Table 8:** Sudden words by study phase.

Phase II (2010-2012)		Phase III (2013-2015)		Phase IV (2016-2017)	
Keywords	Frequency	Keywords	Frequency	Keywords	Frequency
Electronic health record	10	Big data	58	Machine learning	14
Personal health record	9	Mobile health	17	Fog computing	13
DICOM	8	Mobile computing	16	Smart cities	8
Virtualization	7	Ontology	14	Searchable encryption	7
MapReduce	6	Monitoring	13	Virtual reality	6
e-Health	6	Data mining	13	Software	6
PACS^a^	6	Health	13	Research	6
Medical imaging	6	Data security	11	Anonymity	6
Internet	6	PaaS^b^	10	eHealthcare	6
Interoperability	4	Cloud platform	10	Data sharing	6
Access control	4	Mobile cloud	10	Analytics	6
Bioinformatics	4	Body sensor networks	9	Privacy preserving	5
IaaS^c^	4	Optimization	9	Medical image	5
Internet of Things	4	Analysis	9	Distributed systems	5
Cloud security	3	Image processing	9	Biosensors	5

^a^PACS: picture archiving and communication systems.

^b^PaaS: platform as a service.

^c^IaaS: infrastructure as a service.

In Phase III, “big data” was the most frequent sudden word. As aforementioned, the use of big data and other technologies increased in the medical industry during this phase. In addition, “Mobile health,” “mobile computing,” and “mobile cloud” started to emerge. Mobile internet is the product of a combination of mobile communications and the internet. Mobile internet technology is a new technology for high-speed wireless connectivity to mobile devices such as laptops, tablets, and smartphones [61]. Since 2010, the mobile internet has started to change people’s lives and behaviors. With the advancement of mobile internet technology, new terms such as mobile computing technology and mobile health have emerged. Mobile health is the use of mobile internet communication technology to provide health care services such as physical examinations, health care, disease assessments, medical treatment, and rehabilitation. Based on this analysis, mobile health is likely to be an ongoing development trend in the medical industry.

In Phase IV, keywords such as “machine learning,” “fog computing,” and “smart cities” started to appear. As already mentioned, in this phase, machine learning began to be of interest to medical scholars. Machine learning technologies provide effective support for medical data mining to improve the efficiency and quality of data recording and application. The concept of fog computing was first proposed in 2011. Fog computing is a distributed computing infrastructure for the IoT that extends computing power and data analytics applications to the “edge of the network.” It enables customers to gain instant insights through connectivity by analyzing and managing data locally. Tayeb et al [62] summarized the latest research on IoT, cloud computing, and fog computing. These technologies are changing the way we live and work. In 2010, IBM formally proposed the vision of a smart city. A smart city uses information and communication technology to sense, analyze, and integrate the core system of urban operations to respond intelligently to various needs and create a better urban life for mankind. Medical services play a crucial role in the transformation from traditional cities to smart cities. Sajjad et al [63] proposed a method of leukocyte classification and segmentation in microblood smears that not only improves diagnostic accuracy and reduces diagnostic time but also promotes the development of resource-conscious health services in smart cities. Smart cities are bound to become the reality of future cities around the world. With the development of smart cities, the emergence of the proprietary medical term, Wise Information Technology of 120, is bound to lead the future development of the medical industry.

## Discussion

In this study, we conducted a bibliometric analysis using NEVeiwer, SATI, UCINET, and NetDraw of 2878 articles collected from the Web of Science database. The results show that the evolution and development of cloud health care services are closely linked with “Cloud Computing.” The primary keywords were “Cloud Computing,” “Cloud,” “Healthcare,” “Internet of Things,” “Security,” “eHealth,” “big data,” “computing,” “Privacy,” and “mobile cloud computing.” Through the keyword analysis, we summarized the major research hotspots and directions in each phase. In Phase I, most scholars used cloud computing mainly to save costs in the medical field, and the technologies in use were primarily grid computing and cloud computing. In Phase II, scholars began to pay attention to the security of cloud systems, including patient data privacy and security issues. In Phase III, health and medical informatization created big data for health and medical services, and the IoT also developed rapidly. In Phase IV, machine learning and mobile technologies were introduced to the medical field. This study provides not only important references to understand the development of science to inform future research and identify research questions in the field in general but also information to guide practitioners in terms of technological changes and developments in the medical industry. While cloud computing, IoT, and big data have become technical hotspots in this field, electronic health, mobile health, and smart health have developed as main branches.

Emerging information technologies can provide technical support and solve many of the current problems faced by the health care industry as medical data become more heterogeneous, big, and noisy. Through the knowledge evolution analysis, keyword co-occurrence analysis, and sudden word analysis, we identified three trends for the future development of the cloud health care field.

First, cloud computing in health care mainly focuses on saving costs and improving computing efficiency. Some scholars have applied distributed storage and computing to health care systems, demonstrating that cloud computing can enhance the performance of health care systems and increase the satisfaction with smart health care applications and services [43,64,65]. The main issues of cloud computing are focused on distributed storage algorithms, resource-indexing techniques in distributed storage, Hadoop–based distributed storage and computing applications, and distributed computing models. In addition, virtualization of the cloud can reduce the management costs of large data centers and internet-based solutions. It also provides complete user flexibility as well as IT management and control capabilities. Optimized scheduling of resources in the cloud, standardization of cloud computing, and cloud computing security will also be widely used in medical service cloud platforms.

Second, the results show that research hotspots such as “Mobile health,” “mobile computing,” and “mobile cloud” emerged in the second phase. Mobile cloud computing is the combination of mobile internet and cloud computing. The mobile applications of today place higher demands on battery capacity, computing power, storage capacity, and mobile terminal security. Therefore, mobile cloud computing has grown rapidly to meet these needs. Mobile cloud computing technology has been used in health applications to address issues such as limited storage and processing capabilities of mobile devices, interoperability, and availability of electronic medical records [66,67]. In addition, accurate positioning and motion recognition technologies and guaranteed consistent, efficient cloud data will become hotspots in the field of mobile health.

Finally, security and privacy issues always accompany data. With the continuing popularization of emerging technologies, security in the medical field has drawn increasing attention. To ensure the security of cloud health data and the system, there are many challenges to overcome, including big data computing ethics, secure computing in a distributed programming framework, trustworthiness in remote data computing, multigranular access control, and trustworthiness of data sources and data channels.

This study has some limitations. Because cloud health care is an emerging field, the amount of data available for retrieval was relatively limited, which might have impacted the analysis. Second, only keyword evolution was analyzed, and we did not consider other aspects of the evolutionary analysis. These should be addressed in future studies.

## Abbreviations

DICOM: Digital Imaging and Communications in Medicine
IoT: Internet of Things
IT: information technology
NEViewer: Network Evolvement and Trend Detection System
SaaS: software as a service
SATI: Statistical Analysis Toolkit
